# Exploring bias in platelet P2Y_1_ signalling: Host defence versus haemostasis

**DOI:** 10.1111/bph.16191

**Published:** 2023-08-02

**Authors:** Dingxin Pan, Graham Ladds, Khondaker Miraz Rahman, Simon C. Pitchford

**Affiliations:** ^1^ Sackler Institute of Pulmonary Pharmacology, Institute of Pharmaceutical Science King's College London London UK; ^2^ Department of Pharmacology University of Cambridge Cambridge UK; ^3^ Chemical Biology Group, Institute of Pharmaceutical Science King's College London London UK

**Keywords:** aggregation, biased agonism, chemotaxis, haemostasis, inflammation, P2Y_1_, platelets

## Abstract

Platelets are necessary for maintaining haemostasis. Separately, platelets are important for the propagation of inflammation during the host immune response against infection. The activation of platelets also causes inappropriate inflammation in various disease pathologies, often in the absence of changes to haemostasis. The separate functions of platelets during inflammation compared with haemostasis are therefore varied and this will be reflected in distinct pathways of activation. The activation of platelets by the nucleotide adenosine diphosphate (ADP) acting on P2Y_1_ and P2Y_12_ receptors is important for the development of platelet thrombi during haemostasis. However, P2Y_1_ stimulation of platelets is also important during the inflammatory response and paradoxically in scenarios where no changes to haemostasis and platelet aggregation occur. In these events, Rho‐GTPase signalling, rather than the canonical phospholipase Cβ (PLCβ) signalling pathway, is necessary. We describe our current understanding of these differences, reflecting on recent advances in knowledge of P2Y_1_ structure, and the possibility of biased agonism occurring from activation via other endogenous nucleotides compared with ADP. Knowledge arising from these different pathways of P2Y_1_ stimulation of platelets during inflammation compared with haemostasis may help therapeutic control of platelet function during inflammation or infection, while preserving essential haemostasis.

**LINKED ARTICLES:**

This article is part of a themed issue on Platelet purinergic receptor and non‐thrombotic disease. To view the other articles in this section visit http://onlinelibrary.wiley.com/doi/10.1111/bph.v181.4/issuetoc

## Graphical abstract

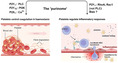

**Abbreviations**
DAMPS: danger‐associated molecular patterns
PKC: protein kinase C

## 1 THE FUNCTIONS OF PLATELETS IN INFLAMMATION COMPARED WITH HAEMOSTASIS

Aggregation is a critical function of platelets to maintain haemostasis within the vasculature, which when deregulated can lead to thrombosis. The activation pathways that lead to platelet aggregation have been extensively researched. To generalize, quiescent circulating platelets bind to endothelial cells via von Willebrand factor (vWF) or collagen at damaged sites of the vessel wall through specific glycoprotein (GPVI‐collagen and GP1b‐vWF) and integrin (α_2_β_1_‐collagen) adhesive interactions. These interactions and that of locally produced thrombin, activate platelets to release mediators such as ADP, 5‐hydroxytryptamine (5‐HT; serotonin) and thromboxane (TXA_2_ ) to further activate platelets and to cause vasoconstriction. In particular, a conformational change occurs to the platelet integrin α_IIb_β_3_ to allow fibrinogen cross‐links to develop with adjacent platelets and thus form an irreversible aggregate or ‘soft’ clot (Mackman et al., 2020).

However, platelets are also critical for the propagation of the inflammatory response to infection and this role can appear to be very distinct from the ability of platelets to undergo aggregation (Middleton et al., 2016; Page & Pitchford, 2013, 2014; Semple et al., 2011; Yeaman, 2014). We and many others have reported the occurrence of these aggregation‐independent platelet functions using experimental models of infection and inflammation induced by a wide variety of stimuli to elicit diverse organ pathologies. In this regard, platelets release granular content of antimicrobial and cytotoxic proteins, reactive oxygen species (ROS) and danger‐associated molecular patterns (DAMPs) and synthesize lipid mediators. Platelets also coordinate the efficient tissue recruitment of granulocytes, monocytes and lymphocytes by acting as a ‘bridge’ during certain adhesion molecule (selectins and integrins) and chemokine‐dependent events of the leukocyte recruitment cascade with the vascular endothelium. Furthermore, platelets communicate via direct cellular interactions with tissue‐resident immune cells (e.g. dendritic cells, macrophages and innate lymphoid cells) to aide antigen processing and recognition to participate in the immune response (Amison, O'Shaughnessy, et al., 2018; de Stoppelaar et al., 2014; Diacovo et al., 1996; Evangelista et al., 1999; Langer et al., 2012; Peters et al., 1999; Pitchford et al., 2003, 2017). However, it is perhaps the accumulation of platelets into tissue in response to inflammatory stimuli, which can occur independently of their intravascular interactions with leukocytes, that serves as a marked example of a non‐thrombotic function of platelets (Boilard et al., 2010; Langer et al., 2012; Pitchford et al., 2008; Shah et al., 2021). While links between haemostasis and inflammation (thrombo‐inflammation) do occur during infection, the separated functions of platelets that propagate the inflammatory response often require motility and adhesion processes to reach and coordinate with tissue‐resident immune cells or kill pathogens within the infected locale. The concept of platelet motility, their ability to migrate towards a chemotactic gradient and to undergo diapedesis across the endothelial barrier, has been a controversial topic in the field of platelet biology compared with the involvement of platelets in intravascular clotting, that has been long established through straightforward observation. The advent of immunohistochemistry, however, allowed researchers to identify and quantify platelets in extravascular tissue compartments in response to infection or subjected to inflammatory stimuli. Thus, a growing number of groups have now reported the ability of platelets to undergo chemotaxis towards a range of chemokines and chemoattractants, using different *in vitro* methodologies, including trans‐migration across endothelial monolayers (Amison, Jamshidi, et al., 2018; Arkless et al., 2023; Czapiga et al., 2005; Fan et al., 2018; Gaertner et al., 2017; Kraemer et al., 2010, 2011; Miao et al., 2020; Nicolai et al., 2020; Palankar et al., 2022; Petito et al., 2018; Pitchford et al., 2008; Schmidt et al., 2011, 2012; Seifert et al., 2022; Shah et al., 2021; Valone et al., 1974; Witte et al., 2021). Fundamentally, this characteristic is by necessity distinct from the action of platelets during haemostasis that induce irreversible adherence to extracellular matrix (e.g. collagen) and contact between adjacent platelets via integrin α_IIb_β_3_ –fibrinogen cross bridges to form an immobile soft platelet ‘clot’. It is noted that there appear to be functions that occur in both contexts of haemostasis and inflammation, for example granular mediator release. It is not understood if shared mechanisms of granule release exist (e.g. P2Y_1_ ‐dependent, see below) or whether differential regulation occurs that may lead to the release of separated cargoes, as demonstrated beyond the context of nucleotide activation (Battinelli et al., 2019; Italiano et al., 2008; Italiano & Battinelli, 2009).

**FIGURE 1:**
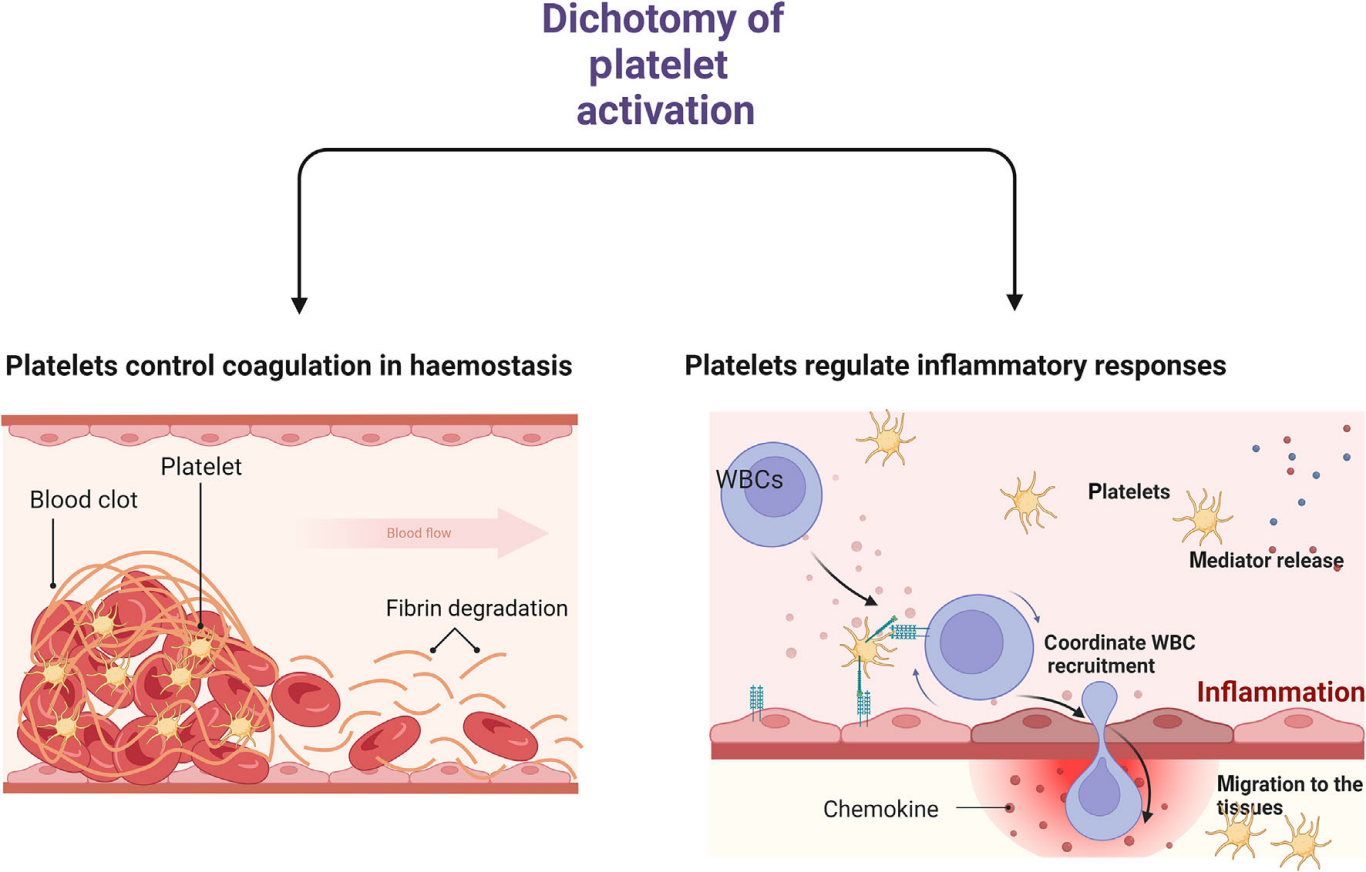
The dichotomy of platelet activation. During haemostasis (left panel), circulating platelets begin to accumulate at the site of injury. They form a platelet plug involving thrombin and fibrin. Platelet activation in haemostasis is key to normal wound repair and healing. During inflammation (right panel), platelet activation is essential for efficient leukocyte (white blood cell [WBC]) recruitment to the sites of inflammation. It also triggers the release of platelet‐derived antimicrobial and proinflammatory mediators, as well as platelet migration. These other processes are non‐thrombotic in nature. Created with BioRender.com.

These critical functions of platelets during inflammation that are fundamentally different to aggregation presumably require the activation of distinct signalling pathways (Figure 1). A concept for a dichotomy in platelet activation was proposed in 1988 to distinguish platelet involvement in thrombotic and non‐thrombotic diseases (Page, 1988). An implication of this concept for a dichotomy (or polytomy) in platelet activation is that drugs developed to target platelet activation in inflammatory diseases will need to be different to current anti‐platelet drugs used for secondary prevention of thrombosis.

**FIGURE 2:**
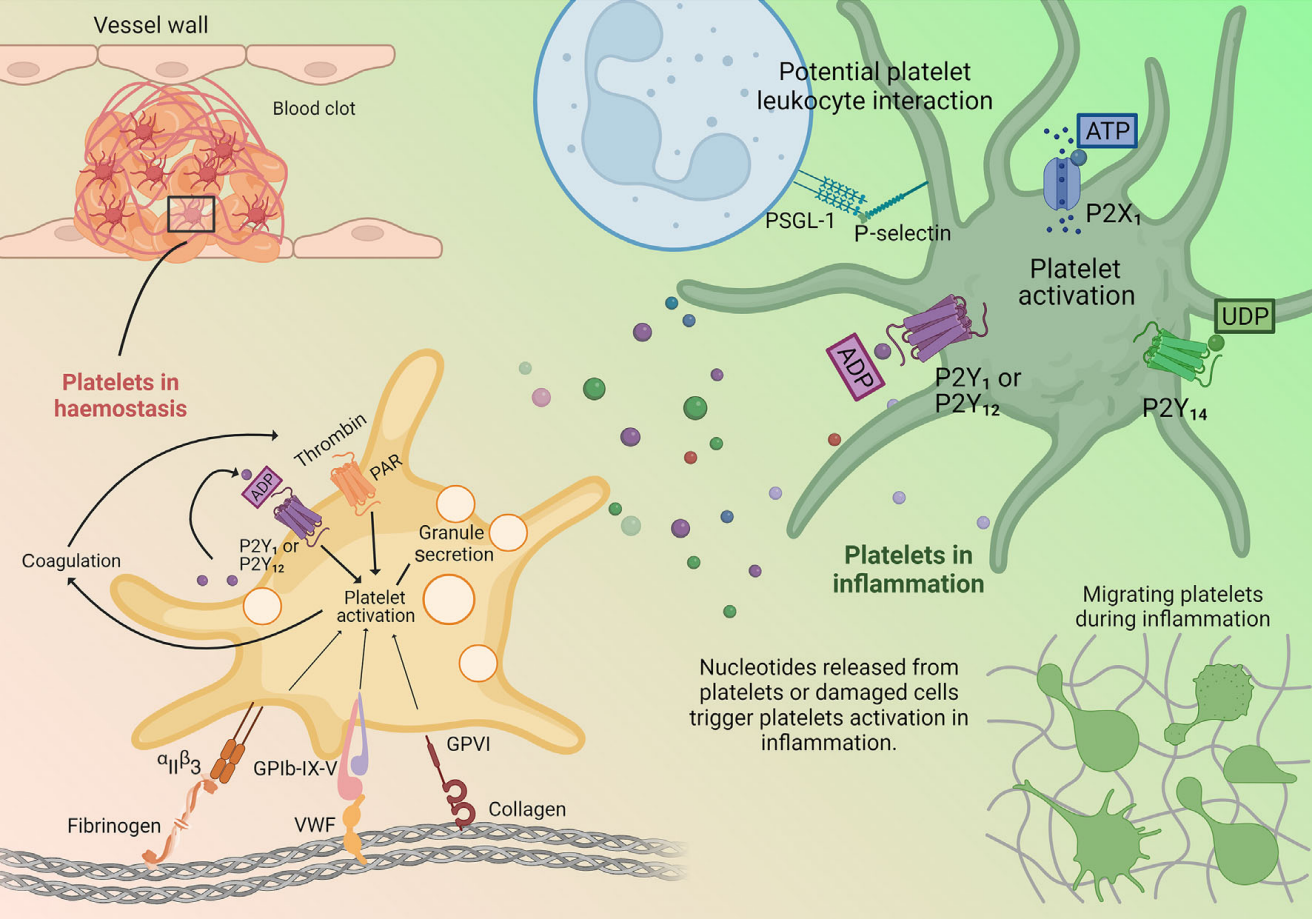
Purinergic nucleotides are key agonists to platelet activation in haemostasis and inflammation. ADP controls platelet aggregation via the P2Y_1_ and P2Y_12_ receptor. ADP, as well as other purinergic nucleotides (ATP and UDP) and nucleosides (adenosine), can act as DAMPs to trigger inflammation in trauma and host defence. The rapid extracellular release and metabolism of nucleotides leads to changes in composition to the ‘nucleotide halo’ that promotes proinflammatory responses, such as platelet‐dependent leukocyte recruitment and platelet motility, rather than coagulation process. Created with BioRender.com.

A requisite component of the immune system response to infection and the propagation of inflammation, is the extravascular release of nucleotides acting as DAMPs. Nucleotides activate inflammatory cells via metabotropic G protein‐coupled P2Y receptors or ionotropic P2X receptors, which are nucleotide‐gated ion channels (Idzko et al., 2014). While originally recognized for their functions in the central nervous system, P2Y and P2X receptors are expressed on cells of the innate and adaptive immune system (Eltzschig et al., 2012; Junger, 2011). The involvement of nucleotides in immune cell activation (secretion of cytokines, proteases and phagocytosis) includes extracellular nucleotide signalling that is essential for the trafficking of immune cells in response to chemoattractants and this involves both autocrine and paracrine signalling events (Ferrari et al., 2016). Using neutrophils as a cell model, Chen et al. (2010) reported how nucleotide signalling is a fundamental mechanism required for inflammatory cell activation and immune defence. Thus, in the case of neutrophils, the formyl peptide receptor 1 (FPR1) and P2Y_2_ receptors were shown to colocalize as tight spatiotemporal associates at the leading edge of the cell. This enabled effective nucleotide signalling via autocrine feedback loops, fed by the release of adenosine triphosphate (ATP) from pannexin‐1 hemichannels activated by FPR1. This process was necessary to amplify the FPR1‐initiated cell response (Chen et al., 2010). Coordination of inflammatory cell function thus required a specialized receptor that detected inflammatory or infectious mediators and a purinergic receptor that then defined and regulated the functional response to such mediators. This concept of nucleotides acting as cofactors to prime cells for signal amplification was further highlighted specifically for cell migration, where nucleotides were necessary to amplify chemotactic signals and direct cell orientation (Chen et al., 2006). Rho‐GTPase (P2Y_2_‐induced) and phosphoinositide 3‐kinase (PI3K) signalling occurred in coordination to provide F‐actin rearrangement and motility. Clearly, directed cell movement requires multiple inputs to control gradient sensing, orientation, traction and retraction (speed). In the context of platelet motility, we have shown that platelets stimulated by chemoattractants N‐formylmethionyl‐leucyl‐phenylalanine (fMLP) and chemokines (stromal cell‐derived factor‐1α [SDF‐1α/CXCL12 α], monocyte chemoattract protein‐1 [MCP1/CCL2] and eotaxin [CCL11]), similarly require the activation of P2Y_1_ via low extracellular concentrations of ADP for platelet‐dependent leukocyte recruitment to the lungs in response to inflammatory stimuli and for platelet motility *in vitro* (Amison et al., 2015; Amison, Jamshidi, et al., 2018; Shah et al., 2021). This supports the concept that nucleotidic cofactor priming for signal amplification is a generally required mechanism for inflammatory functions (Figure 2).

However, the composition of purinergic nucleotides (ATP, ADP and uridine diphosphate [UDP]) and nucleosides (adenosine) that act as DAMPs surrounding cells during trauma and host defence is potentially diverse. In particular, the rapid extracellular release and metabolism of nucleotides leads to changes in the composition of the extracellular milieu of nucleotides. This process has been coined by others as a ‘nucleotide halo’ to describe that nucleotide levels are tightly controlled to perform both synergistic and repressive actions during inflammation and haemostasis, and cellular activation by nucleotides needs to be considered in the ‘whole’ rather than emphasis on any particular nucleotide (Antonioli et al., 2019; Burnstock & Boeynaems, 2014; Trautmann, 2009). Platelets express four P2 receptors (ATP‐gated P2X_1_ ion channel and the G protein‐coupled receptors [GPCRs] P2Y_1_ and P2Y_12_ —both activated by ADP, P2Y_14_ —activated by UDP‐glucose) and two P1 receptors:‐ the adenosine activated GPCRs A_2A_ and A_2B_ receptors (Gachet, 2006; Jacobson et al., 2011; Wolska & Rozalski, 2019). The relevance of P2Y_14_ activation of platelets has not been extensively described, but P2Y_14_ appears to lack an involvement in platelet aggregation, while preliminary data suggest P2Y_14_ might be involved in platelet‐associated neutrophil migration (Amison et al., 2017; Dovlatova et al., 2008). Elsewhere, a direct role for P2Y_14_ on leukocyte activation has been reported (Sesma et al., 2012, 2016). A_2A_ and A_2B_ receptors stimulation leads to inhibition of platelet activation and aggregation (Wolska & Rozalski, 2019). Platelet activity therefore has the potential to be regulated in a multi‐faceted fashion by the ‘nucleotide halo’ during inflammation and we discuss our emerging understanding of the role of platelet P2Y_1_ in this context compared with haemostasis (Figure 2).

## 2 PLATELET P2Y_1_ FUNCTION IN HAEMOSTASIS

**FIGURE 3:**
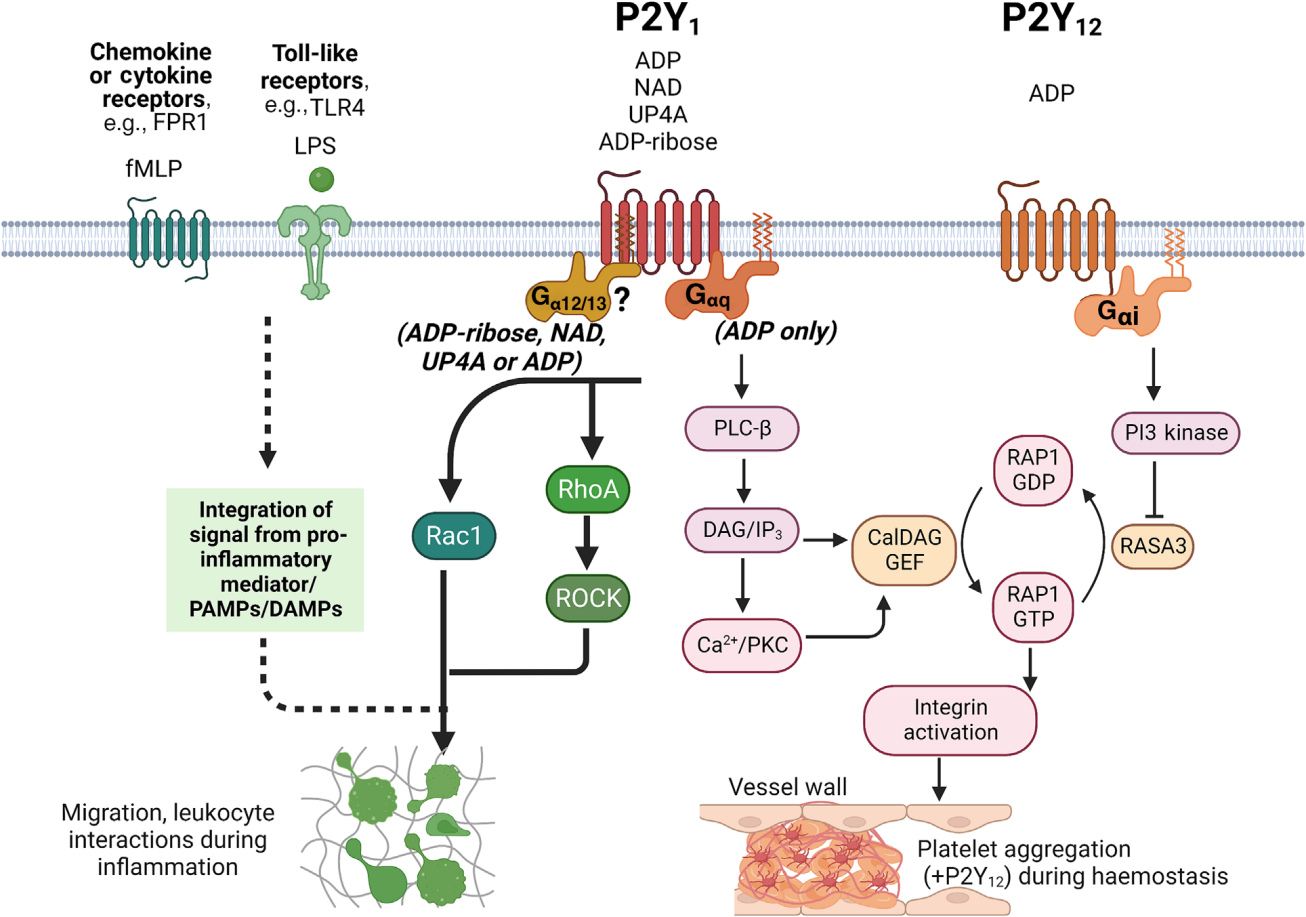
Platelet P2Y_1_ activation displays alternative and biased signalling pathways. Classical platelet activation in haemostasis induces P2Y‐dependent shape change, granule secretion and aggregation via the canonical PLC (P2Y_1_) and PI3K (P2Y_12_) signalling pathways. In inflammation, P2Y_1_ activation on platelets promotes platelet motility, adhesion and interaction with leukocytes. Such activation by inflammatory stimuli requires the non‐canonical small GTPases Rac1 and RhoA downstream of P2Y_1_ receptor signalling. The molecular interaction required with the P2Y_1_ receptor to allow these signalling processes to occur is not known, but in other systems, this has been shown to be G_12/13_ dependent. Created with BioRender.com.

ADP activates platelets by signalling through P2Y_1_ and P2Y_12_. P2Y_1_ is coupled to the G_αq/11_ family of G proteins, which activates PLCβ resulting in intracellular calcium ion (Ca^2+^_i_) mobilization and protein kinase C (PKC) activation. The RAS‐related protein guanine‐nucleotide exchange factor (RAP‐GEF) calcium and diacylglycerol‐regulated guanine‐nucleotide exchange factor 1 (CalDAG‐GEFI or RASGRP2) contain binding sites for Ca^2+^ and DAG, and are the main Ca^2+^_i_ sensor induced by many platelet agonists, including ADP (Stefanini et al., 2009). CalDAG‐GEFI is critically required for aggregation, because the GEF domain catalyses the activation of the small guanosine triphosphate hydrolase (GTPase) ras‐related protein 1b (RAP1b) to promote transient integrin α_IIb_β_3_–fibrinogen crosslinking of adjacent platelets (Crittenden et al., 2004; Lova et al., 2002; Stefanini & Bergmeier, 2016). Furthermore, Ca^2+^‐dependent TXA_2_ generation and PKC activation pathways stimulate granule release leading to increased extracellular ADP. Subsequent ADP activation of P2Y_12_ (G_αi/o_) then drives a secondary wave of stable platelet activation because PI3K signalling inhibits the opposing RAP‐GTPase‐activating protein (RAP‐GAP) RASA3 that acts as a constitutive molecular brake to CalDAG‐GEFI (Stefanini et al., 2015). Thus, synergy occurs with P2Y_1_ (or other G_αq/11_ GPCRs) to produce sustained RAP1 activation (Stefanini et al., 2015). Morphologically, this specific role of P2Y_1_ in early (primary phase) activation by ADP is therefore displayed by platelet shape change to form filopodia and granule release (Leon et al., 1999), these being necessary physiological events that precede full (secondary phase) ADP‐induced platelet aggregation by P2Y_12_ (Baurand et al., 2001; Fabre et al., 1999; Gachet, 2006; Leon et al., 1999; Storey et al., 2000) (Figure 3). In this context, ADP acts as a cofactor for platelet activation by thrombin or collagen, and amplifies the responses caused by other weak agonists such as 5‐HT and adrenaline. Both P2Y_1_ and P2Y_12_ have been reported to have the same sensitivity to ADP (potency between 1 and 2 μM) and likely to be activated simultaneously *in vivo* (Daniel et al., 1998; Jin & Kunapuli, 1998). Interestingly, interpretation of Ca^2+^_i_ mobilization patterns has provided a more nuanced understanding of spatiotemporal synergy between P2Y_1_ and P2Y_12_. Transient Ca^2+^_i_ waves downstream of P2Y_1_ invoked α_IIb_β_3_ activation, but this only occurred when P2Y_12_ was co‐stimulated. Further, the extent of fibrinogen binding was dependent on the amplitude and periodicity of Ca^2+^_i_ waves induced by both receptors (Bye et al., 2020). Platelets express approximately 150–200 P2Y_1_ receptors per cell (Baurand et al., 2000) and this is considered a low number compared with other GPCRs. However, reciprocal signalling between P2Y_1_ and P2Y_12_ reveals checkpoints (e.g. tyrosine protein kinase [Src kinase] and G protein‐coupled receptor kinase 2/beta adrenergic receptor kinase 1 [GRK2]) that balance platelet activation during normal haemostasis and thus, the interplay between these two receptors is defined as more than uni‐directional or comparative receptor density. Further, there is a need for operational balance after nucleotide activation (Hardy et al., 2004, 2005; Zhao et al., 2022). Cooperativity can also occur between P2Y_1_ and the ATP‐gated ion channel P2X_1_ (Jones et al., 2014; Tolhurst et al., 2005; Vial et al., 2002). Lastly, P2Y_1_ activity can also be controlled via a PKC‐dependent receptor desensitization (internalization) process (Mundell et al., 2006).

The creation of highly selective P2Y_1_ antagonists (e.g. MRS2179, MRS2279 and MRS2500) have proved to be potent at inhibiting platelet aggregation *in vitro* and suppressing thrombosis in animal models. Highlighting the P2Y_1_ receptor as an appropriate pharmacological target to control platelet activation for patients at risk of secondary cardiovascular events (Baurand et al., 2001; Boyer et al., 1998; Cattaneo et al., 2004; Hechler et al., 1998, 2006; Housten et al., 1998; Kim et al., 2003). The short half‐life and limited bioavailability of these antagonists has led to the development of compounds with a non‐nucleotide structure that could be orally administered, thus demonstrating the potential for P2Y_1_ to be a ‘druggable’ target (Costanzi et al., 2012; Morales‐Ramos et al., 2008).

While signalling via PLCβ is considered the canonical pathway by which P2Y_1_ activates platelets. ADP can also activate the small Rho‐GTPases Ras homolog family member A (RhoA, acting on Rho‐associated coiled‐coil containing protein kinase 1: ROCK1), Ras‐related C3 botulinum toxin substrate 1 (Rac1) and cell division control protein 42 homolog (cdc42) (Aslan & McCarty, 2013; Beck et al., 2017) that are otherwise necessary to work in a coordinated and complementary fashion for the adhesion, activation and motility of leukocytes (Lawson & Burridge, 2014). In the context of platelets, it is believed that Rho‐GTPases, combined with PLC‐induced Ca^2+^_i_ mobilization, are responsible for shape changes to occur that would be necessary for filopodia and lamellipodia formation. This results in platelet spreading at the onset of aggregation, whereby Ca^2+^ mobilization initiates the phenomena, and RhoA then maintains the shape change over time (Eckly et al., 2001; Jin et al., 1998; Paul et al., 1999; Wilde et al., 2000). Furthermore, P2Y_1_ was reported to be involved in this RhoA‐dependent platelet change (Wilde et al., 2000), yet this pathway was considered to be insignificant at concentrations of ADP sufficient to cause platelet aggregation, because the presence of a Rho‐kinase inhibitor (Y27632) had no effect on ADP‐induced Ca^2+^_i_‐dependent shape change (Bauer et al., 1999). While it is recognized that platelet Rho‐GTPase activities do underlie platelet function in haemostasis as a multifactorial process involving many Rho‐GTPase family members and regulatory proteins (Aslan, 2019), ultimately RhoA activity alone is insufficient for full platelet aggregation in response to ADP (Amison, Jamshidi, et al., 2018; Soulet et al., 2005). Furthermore, ADP‐induced Rac1 activation by specific guanine‐nucleotide exchange factors (Rac‐GEFs) also appears to play minor roles in full aggregation (Amison, Jamshidi, et al., 2018; Aslan et al., 2011; Pearce et al., 2007; Pleines et al., 2012). Yet it is possible that Rho‐GTPases are involved in the shape change of platelets for non‐aggregatory functions, for example, cellular shape change and polarity required for adhesion and motility events during inflammation, ROS production or bacterial scavenging (Aslan, 2019; Gaertner et al., 2017).

## 3 P2Y_1_ ACTIVITY IN INFLAMMATION AND PLATELET FUNCTION

Purinergic nucleotides have received much attention as DAMPs during trauma and host defence, controlling leukocyte activation, adhesion molecule expression and motility (Chen et al., 2006; Ferrari et al., 2016). Various studies have reported a role for P2X_1_, P2Y_1_ and P2Y_12_ receptors in inflammation, but to focus on P2Y_1_, a global participation is evident in models of bacterial infection (Geary et al., 2005), allergic lung inflammation (Gao & Gao, 2021), LPS‐induced lung inflammation (Amison et al., 2017) and inflammatory bowel disease (Zhang et al., 2020). Other studies have reported a specific role for the P2Y_1_ receptor to activate platelets (but interestingly not platelet P2Y_12_ or P2X_1_ in animal models where inflammation is not associated with changes in haemostasis) in the context of platelet‐dependent leukocyte (eosinophil and lymphocyte) recruitment in allergic airways inflammation and LPS‐induced lung neutrophilia (Amison et al., 2015, 2017). In these contexts, the platelet‐dependent pulmonary recruitment of leukocyte requires expression of platelet selectin (P‐selectin/CD62) or P‐selectin glycoprotein ligand‐1 (PSGL‐1) (Kornerup et al., 2010; Pitchford et al., 2003, 2005), and it is interesting that the formation of platelet–leukocyte complexes and platelet‐dependent leukocyte chemotaxis have been shown to be P2Y_1_ dependent (Amison et al., 2015, 2017; Anderson et al., 2020; Badrnya et al., 2012). While platelet P‐selectin expression by ADP requires P2Y_1_ stimulation (Anderson et al., 2020), this can be mediated by either PLC or RhoA and Rac1 (Akbar et al., 2007, 2016; Anderson et al., 2020). Other studies of non‐thrombotic functions of platelets that are P2Y_1_ dependent include platelet killing of parasites (McMorran et al., 2009), chemokine CXCL16‐mediated platelet adhesion to inflamed endothelium (Borst et al., 2012) and platelet chemotaxis (Amison, Jamshidi, et al., 2018; Arkless et al., 2023). Consequently, the activation of platelets by P2Y_1_ in non‐thrombotic diseases may lead to diverse functional outputs and is worthy of exploration in other inflammatory settings (Figure 2).

Understanding how the platelet P2Y_1_ receptor becomes activated in response to immune and inflammatory stimuli, and the downstream signalling events that might occur, has been compared with P2Y_1_ signalling to induce platelet aggregation during haemostasis. Data implicated that signalling by the Rho‐GTPase RhoA downstream of platelet P2Y_1_ activation controls pulmonary leukocyte recruitment during airway inflammation (Amison et al., 2015). We also investigated the role of another small GTPase, Rac1 in platelets, again using diverse models of both allergic and non‐allergic airway inflammation. We showed that the expression of specific direct activators of Rac1 (Rac‐GEFs) in platelets are also required for leukocyte recruitment (Pan et al., 2015).

Furthermore, the use of *in vitro* P2Y_1_‐dependent inflammatory function assays (platelet motility and platelet‐induced neutrophil chemotaxis) was compared with ADP‐induced platelet aggregation and showed that Rho‐GTPase (RhoA, Rac1), but not PLC signalling was necessary for these non‐thrombotic platelet functions. Whereas, PLC but not Rho‐GTPase signalling was necessary for ADP‐induced platelet aggregation (Amison, Jamshidi, et al., 2018) (Figure 3). Thus, there appear to be requisite roles for Rho‐GTPase signalling in response to P2Y_1_ activation in the non‐thrombotic functions of platelets. In these contexts, it is not yet understood how P2Y_1_‐desensitization will occur if there is an absence of PKC signalling (Mundell et al., 2006) or whether persistent stimulation of the receptor occurs.

The activation and function of platelets in the context of asthma has been reported extensively, as an example of a non‐thrombotic disease. In particular, clinical studies demonstrate platelet activation and tissue recruitment after allergen challenge in patients with asthma, and this is not associated with heightened haemostasis *in vivo* but instead the presentation of impaired platelet aggregation to ADP and other agonists *ex vivo* (Gresele et al., 1993; Ind, 1991; Kowal et al., 2006; Maccia et al., 1977; Szczeklik et al., 1986). Yet animal studies repeatedly demonstrate that platelet activation in the context of allergic airways inflammation is causal and requisite to the development of the inflammatory response (see reviews: Page & Pitchford, 2014; Takeda et al., 2018). Despite high levels of nucleotides (ATP and ADP) (Gao & Gao, 2021; Idzko et al., 2007) being released in the lungs during allergic inflammation, at concentrations presumably sufficient for platelet aggregation (20–30 μM) (Tymvios et al., 2008), it is not understood why a heightened platelet aggregation does not occur in parallel to their raised inflammatory function that would be both P2Y_1_ dependent (Amison et al., 2015). Studies focusing on P2Y_1_‐induced platelet activation tend to be based on responses to ADP. However, other endogenous nucleotides have also been reported to act as P2Y_1_ agonists and are found extracellularly (Del Principe et al., 1986; Durnin et al., 2014; Hwang et al., 2011; Lüthje & Ogilvie, 1984). Some of these nucleotides (ADP‐ribose and the dinucleotides uridine adenosine tetraphosphate [Up4A] and nicotinamide adenine dinucleotide [NAD^+^]) mimic the ability of ADP to induce platelet chemotaxis via P2Y_1_ activation, and RhoA and Rac1 signalling, but unlike ADP other nucleotides are unable to cause platelet aggregation via PLC activity (Arkless et al., 2023) (Figure 3). While the physiological relevance of these findings is not known, some of these nucleotides acting on the P2Y_1_ receptor outwardly display properties, whereby they have a preference or bias towards one pathway over another. This concept of agonist‐induced signalling bias ‘agonist bias’ has been described for many different GPCRs and is thought to arise due to GPCRs being able to adopt an ensemble of different conformations, depending upon the stimulating agonist that bring can then produce different signalling events (Kenakin, 2012). Such phenomena might therefore influence platelet function within the milieu of the nucleotide ‘halo’ during inflammation. However, the molecular details for agonist bias by different nucleotides acting on platelet P2Y_1_ receptor to selectively stimulate a subset of signalling pathways have not yet been defined.

## 4 P2Y_1_ SIGNALLING AND OCCURRENCE OF AGONIST BIAS

With the advent of phosphoproteomic technology, a temporal understanding of signalling events has been illustrated following the activation of platelets by ADP (Beck et al., 2017). This study showed over 600 regulated events occurred (from approximately 4800 tested) and coalesced into an order of critical hubs of signalling pathways, revealing the complexity and also the diversity of signalling events that occur after ADP activation in platelets (Beck et al., 2017). While the principal G protein coupling of P2Y_1_ receptor is G_αq/11_ (required for PLCβ/inositol trisphosphate [IP_3_]/diacylglycerol [DAG] cascade to increase subsequent Ca^2+^_i_ concentration and activation of PKC, as part of the platelet aggregation process), there is potential for promiscuity for P2Y_1_ receptor G protein interactions that may account for the existence of these diverse signalling pathways. The four main families of G proteins (G_s_, G_i/o_, G_q/11_ and G_12/13_) exist with 20 different G_α_ subunits. Different G_α_ subunit couplings might therefore dictate GCPR downstream signalling pathways after receptor stimulation (Inoue et al., 2019). Aside from coupling to the G_αq/11_ subunit, P2Y_1_ has been reported (using *in vitro* bioluminescent resource energy transfer [BRET] biosensors) to display considerable G protein promiscuity, activating members of all four G protein families (G_s_, G_i/o_, G_q/11_ and G_12/13_), in addition to potential signalling from the recruitment of β‐arrestin (Gao & Jacobson, 2017; Inoue et al., 2019). The potential for selective responses by P2Y_1_ agonists was demonstrated comparing the structurally distinct nucleotides 2MeADP and MRS2365 with the dinucleotide diadenosine tetraphosphate (Ap4A), on GTPγS binding, β‐arrestin2 recruitment and ERK1/ERK2 stimulation. Ap4A behaved as a partial agonist in GTP*γ*S binding, β‐arrestin2 recruitment and β‐arrestin2‐mediated extracellular signal‐regulated kinase (ERK1/2) stimulation but is a full agonist in G_q/11_‐mediated ERK1/2 stimulation, in contrast to 2MeADP and MRS2365 which acted as full agonists in each assay (Gao & Jacobson, 2017). Additional potential effects of G protein promiscuity may therefore add to the quality and quantity of agonist responses to P2Y_1_, signalling selectivity and the possibility for agonist bias.

The structure of the P2Y_1_ receptor reveals it has distinctive orthosteric binding sites (Zhang et al., 2015), which has allowed the binding of the orthosteric antagonist (MRS2500) and allosteric modulator (BPTU) to be evaluated (Yuan et al., 2016). There is considerable helix movement of agonist bound P2Y_1_, with structural plasticity in the transmembrane positions and extracellular loops, and this may confer potential qualitative differences in activation after agonist binding (Zhang et al., 2015). We previously reported that structurally different agonists to P2Y_1_ (the synthetic agonist MRS2365 compared with ADP) display comparatively different efficacies in functional assays used to differentiate platelet activation in haemostasis (aggregation) to inflammation (chemotaxis), despite being used at optimal concentrations (Amison, Jamshidi, et al., 2018). Interestingly, the endogenous nucleotide ADP interacted with different amino acid residues within the orthosteric binding site compared with MRS2365 (Amison, Jamshidi, et al., 2018). Subsequently, when comparing the docking of four endogenous nucleotides ADP, NAD^+^, ADP‐ribose and Up4A, which behaved qualitatively differently in the P2Y_1_‐dependent aggregation (requiring PLCβ activity) and chemotaxis (requiring Rho‐GTPase activity) assays, *in silico* analysis revealed that while ADP interacts with about 10 different amino acids within the P2Y_1_ binding site (and uniquely with ASN283) and sat deep in the binding pocket, the other nucleotides (NAD^+^, ADP‐ribose and Up4A) had additional contacts with other amino acids within the binding pocket, in a shallower position. Thus, unique patterns of amino acid interaction distinguish the non‐biased P2Y_1_ agonist ADP to nucleotides (NAD^+^, ADP‐ribose and Up4A) that demonstrated biased agonist properties with respect to P2Y_1_‐dependent platelet function, and the selective interaction with different amino acids within the binding pocket might play an important role in the observed bias for some endogenous nucleotide ligands (Arkless et al., 2023).

The molecular mechanism for agonist‐biased activities of the P2Y_1_ in platelets may arise from a number of different routes. First, *in vitro* assays suggest P2Y_1_ to be highly promiscuous in its coupling to G proteins families (e.g., G_12/13_‐Rho‐GTPases); second, P2Y_1_ is known to recruit both beta adrenergic receptor kinase 1 (GRK2) and β‐arrestin2 in agonist‐dependent manner both of which could lead to contrasting signalling outcomes. Third, the dynamics of both agonist binding and receptor internalization can lead to changes in downstream signalling dependent upon the stimulating agonist, and finally, bias can arise from alternative effectors that are responsible for PLCβ‐independent G_αq/11_ functions reviewed elsewhere (Sánchez‐Fernández et al., 2014). In this regard, p63Rho‐GEF has been shown to provide a link between G_αq/11_‐coupled GPCRs and RhoA activation (Lutz et al., 2007) The relationship between PLCβ and p63Rho‐GEF has been described as competitive for G_αq/11_ and might provide exclusive, as well as alternative signalling (Lutz et al., 2005; Sánchez‐Fernández et al., 2014). Another Rho‐GEF (Trio) has also been reported as necessary for Gαq‐mediated RhoA and Rac1 signalling (Vaqué et al., 2013). These may not be relevant to the activation of platelets via P2Y_1_ and Rho‐GTPase activity, but they show that exclusive and non‐PLCβ signalling pathways exist.

## 5 IMPLICATIONS AND OPPORTUNITIES FOR DRUG DEVELOPMENT

P2Y_1_ receptors are promising therapeutic targets because effects can be produced rapidly without the need for changes in nuclear transcription or translation (especially because platelets are anucleate), compared with established anti‐inflammatory drugs, for example, glucocorticosteroids (Pitchford et al., 2019). Additionally, the short platelet lifespan and their involvement in multiple levels of the immune response mean that the chances of operational redundancy of novel therapeutics that target platelets are reduced (Pitchford et al., 2019). Currently, P2Y_1_ antagonists act in a ‘balanced’ manner, blocking platelet activation by binding to the orthosteric site of the receptor. Pharmacologically, P2Y_1_ antagonists (e.g. MRS2500 and MRS2179) have been reported to effectively supress inflammation induced from different mediators in animal models of disease (Amison et al., 2015, 2017). When administered prophylactically, the level of inhibition of inflammatory parameters observed with MRS2500 and MRS2179 is substantial (80% to 90% inhibition) and this is notable given these compounds do not have favourable pharmacokinetic characteristics for sustained *in vivo* effects (Baurand et al., 2001; Hechler et al., 2006). These pharmacological studies would benefit from use of reciprocal P2Y_1_ transgenic models to better study the dependency of platelet P2Y_1_ over the continuum of an inflammatory response. However, compounds such as MRS2500 and MRS2179 have been developed to address the role of P2Y_1_ receptors in haemostasis; hence they impair ADP‐induced platelet aggregation and platelet‐induced leukocyte activation or platelet motility and therefore generally affect haemostasis and inflammation. The exploitation of the biased P2Y_1_ signalling pathway using chemical tools will usher new drug discovery research aimed at differentiating the distinct and competing aggregatory and inflammatory functions of P2Y_1_ receptor. This could lead to the development of functionally selective P2Y_1_ antagonists as new generation anti‐inflammatory drugs that can be used to treat diseases like asthma, chronic obstructive pulmonary disease (COPD) or pneumonia with significant unmet clinical need. These drugs will be distinctly different compared with the currently available anti‐platelet drugs that are used for the treatment and secondary prevention of thrombosis (Mackman et al., 2020; Xiang et al., 2019). The availability of new chemical scaffolds that can target the biased pathways, biochemical experiments capable of differentiating biased responses and the availability of relevant animal models will be the key to succeed in this exciting but challenging endeavour.

### 5.1 Nomenclature of targets and ligands

Key protein targets and ligands in this article are hyperlinked to corresponding entries in the IUPHAR/BPS Guide to PHARMACOLOGY http://guidetopharmacology.org and are permanently archived in the Concise Guide to PHARMACOLOGY 2021/22 (Alexander, Christopoulos, et al., 2021; Alexander, Fabbro, et al., 2021; Alexander, Kelly, et al., 2021).

## AUTHOR CONTRIBUTIONS

Dingxin Pan, Graham Ladds and Khondaker Miraz Rahman wrote the article and provided critical commentary and revision. Simon C. Pitchford proposed and conceptualized the review and also wrote the article. Figures are created with BioRender.com, with permission from Professor Clive Page.

## CONFLICT OF INTEREST STATEMENT

No author has a conflict of interest to disclose.
